# Cardiac ischemia modulates white adipose tissue in a depot-specific manner

**DOI:** 10.3389/fphys.2022.1036945

**Published:** 2022-10-28

**Authors:** Luzhou Wang, Heba Zabri, Simone Gorressen, Dominik Semmler, Christian Hundhausen, Jens W. Fischer, Katharina Bottermann

**Affiliations:** Institute for Pharmacology, Medical Faculty and University Hospital Düsseldorf, Heinrich-Heine-University Düsseldorf, Düsseldorf, Germany

**Keywords:** Myocardial infarction, white adipose tissue depots, lipolysis, browning, adipokines, lipogenesis

## Abstract

The incidence of heart failure after myocardial infarction (MI) remains high and the underlying causes are incompletely understood. The crosstalk between heart and adipose tissue and stimulated lipolysis has been identified as potential driver of heart failure. Lipolysis is also activated acutely in response to MI. However, the role in the post-ischemic remodeling process and the contribution of different depots of adipose tissue is unclear. Here, we employ a mouse model of 60 min cardiac ischemia and reperfusion (I/R) to monitor morphology, cellular infiltrates and gene expression of visceral and subcutaneous white adipose tissue depots (VAT and SAT) for up to 28 days post ischemia. We found that in SAT but not VAT, adipocyte size gradually decreased over the course of reperfusion and that these changes were associated with upregulation of UCP1 protein, indicating white adipocyte conversion to the so-called ‘brown-in-white’ phenotype. While this phenomenon is generally associated with beneficial metabolic consequences, its role in the context of MI is unknown. We further measured decreased lipogenesis in SAT together with enhanced infiltration of MAC-2^+^ macrophages. Finally, quantitative PCR analysis revealed transient downregulation of the adipokines adiponectin, leptin and resistin in SAT. While adiponectin and leptin have been shown to be cardioprotective, the role of resistin after MI needs further investigation. Importantly, all significant changes were identified in SAT, while VAT was largely unaffected by MI. We conclude that targeted interference with lipolysis in SAT may be a promising approach to promote cardiac healing after ischemia.

## Introduction

Myocardial infarction (MI) is a pathology with strong systemic implications. Due to various factors as reduced cardiac output, pain and distress as well as dying cardiomyocytes, catecholamine levels during and after cardiac ischemia are elevated (Valori et al., 1967). This systemic adrenergic stimulation does not only increase cardiac performance and blood pressure, but also impacts several other organs as kidney, liver and adipose tissue. Catecholamine stimulation is one of the strongest stimuli for peripheral adipose tissue lipolysis, the process of hydrolysis of triacylglycerides (TAG) into glycerol and fatty acids (Schreiber et al., 2019). It is long known that circulating levels of free fatty acids (FFA) are elevated in plasma samples of patients with myocardial infarction (Vetter et al., 1974) and this could also be shown in small animal models of myocardial infarction (Yue et al., 2003). High levels of FFA are known to be detrimental to the ischemic myocardium (Essop and Opie, 2020). The first and rate limiting enzyme of lipolysis is *adiposetriglyceride lipase* (ATGL) (Haemmerle et al., 2006) which is activated *via* catecholamine-induced stimulation of PKA and release of its cofactor CGI-58 from Perilipin 1. It catalyzes the hydrolysis from TAGs to DAGs and free fatty acids. Adipocyte ATGL is a major player of whole-body lipid metabolism and glucose homeostasis (Schreiber et al., 2019) and has been shown to be involved in cardiac pathologies as pressure overload (Parajuli et al., 2018; Salatzki et al., 2018; Bottermann et al., 2022), catecholamine-induced heart failure (Takahara et al., 2021; Thiele et al., 2021) or myocardial infarction (Bottermann et al., 2020).

Next to its well-known function in the storage and provision of energy, adipose tissue is more and more recognized also as an important endocrine organ which secretes a plethora of factors as adipokines, cytokines, micro-RNAs (Thomou et al., 2017) or extracellular vesicles (EV) (Crewe et al., 2021; Li et al., 2021). Several of these adipose tissue derived factors were also discovered to be involved in cardiac remodeling after myocardial infarction, for example adiponectin (Shibata et al., 2007), miR30d (Li et al., 2021) or small EVs (Crewe et al., 2021).

White adipose tissue consists of several depots throughout the body, which can roughly be divided into visceral and subcutaneous white adipose tissue (VAT and SAT) and are associated with different cardiovascular risks (Oikonomou and Antoniades, 2019). Visceral WAT mainly surrounds the inner organs (pericardial, perigonadal, retroperitoneal, omental, mesenteric) and is negatively correlated with cardiovascular risk. Subcutaneous WAT is composed of inguinal, gluteal and abdominal depots and is considered to be protective in the context of cardiovascular disease (Wronska and Kmiec, 2012; Chusyd et al., 2016). Visceral and subcutaneous WAT differ with respect to adipocyte size and number, adipokine secretion and lipid storage and release capacity. In mice SAT adipocytes are smaller than VAT adipocytes, however the overall adipocyte number is higher in SAT. A simplified view is that VAT seems to be more responsible for energy storage and release, while SAT seems to be the major source of adipokines as leptin and adiponectin (Wronska and Kmiec, 2012; Schoettl et al., 2018). However, this strongly depends on species and metabolic state.

Due to its function as regulator of whole body metabolism and its secretory activity, targeting of adipose tissue in cardiac pathologies is seen as a promising therapeutic approach (Smeir et al., 2021). However, lipolysis in response to MI seems to be activated only transiently as catecholamine and free fatty acid levels are upregulated within the first hours after myocardial ischemia and found to be reduced thereafter (Oliver, 2014). It remains unclear, how myocardial ischemia and the post-ischemic remodeling processes impact white adipose tissue and if the main WAT depots are affected differentially, which could open up new therapeutic strategies.

We hypothesized that myocardial ischemia induces acute and chronic changes within subcutaneous (inguinal) and visceral (gonadal) WAT at different timepoints of reperfusion and therefore examined in the present study key features of white adipose tissue such as morphology, inflammation and gene expression.

## Materials and methods

### Animals

12 weeks old C57Bl/6J male mice (Janvier Labs) underwent 60 min closed chest ischemia (Nossuli et al., 2000) followed by 24 h, 7 days or 28 days of reperfusion (I/R). For induction of closed chest ischemia, mice received surgery 5–7 days before ischemia to introduce a suture around the left ventricular descending artery (LAD). Mice were anesthetized with 90 mg/kg BW Ketamin/15 mg/kg BW Xylazin, intubated and mechanically ventilated. The chest was opened in the 3rd intercostal space, the LAD exposed and a 7/0 prolene suture passed underneath the LAD. A small PE-10 tube was threaded on both ends of the suture, loosely forming a loop around the LAD. The ends of the suture were left in a subcutaneous skin pocket and the ribs and skin closed. Mice were allowed to recover from surgery for 5–7 days and then anesthetized with 2% isoflurane, and ischemia was induced under ECG control by using 5 g weights at each end of the suture. Body temperature was maintained at 37.5°C. Postoperative analgesia was achieved using buprenorphine (0.05 mg/kg BW). Blood was sampled from the tail vein before ischemia and after 30 min of reperfusion. Echocardiography was performed with ultrasound device Vevo 3100 (Visual Sonics) and ultrasound probe MX400. All experiments were in accordance with the local animal regulations and approved by the local authorities (LANUV NRW, AZ: 81-02.04.2019.A397).

After the intended reperfusion times mice were sacrificed and inguinal and gonadal fat pads, as representative for subcutaneous and visceral WAT, excised. For both depots, the right pad, used for protein isolation, and half of the left pad, used for RNA isolation, were briefly washed in cold PBS and snap frozen in liquid nitrogen. The other half of the left pad, used for histological analysis, was 4% formalin fixed overnight and paraffin embedded.

### Non-esterified fatty acid measurement

NEFA measurement was performed in serum using Wako HR NEFA Kit (Fujifilm) according to the manufacturers’ recommendation. Briefly, a standard series composing of 0, 0.125, 0.25, 0.5, and 1 mM NEFA Standard was prepared in advance. 4 µl serum or standard and 200 µl FUJIFILM NEFA (HR) 1 were incubated for 5 min at RT, samples were measured once by SYNERGY microplate reader at a wavelength of 550 nm and 100 µl NEFA (HR) 2 was added, further incubated for another 5 min at RT and measured again at a wavelength of 550 nm.

### Hematoxylin and eosin (H&E) staining

5 µm paraffin sections were deparaffinized, incubated with hematoxylin (Sigma-Aldrich) for 1 min, followed by a short rinse in tap water and 1% HCl. Bluing was performed under running tap water for 10 min. Afterwards, 1% eosin solution (Carl Roth) was incubated for 1 min, sections were dehydrated and mounted with Roti®-Mount (Carl Roth).

### Quantification of cell size

Samples were analyzed by microscope Zeiss Imager.M2 using a 10× objective. The size of 200 cells was measured for each animal using ImageJ (Rasband, W.S., ImageJ, U. S. National Institutes of Health, Bethesda, Maryland, United States, https://imagej.nih.gov/ij/, 1997–2018) by manually drawing the outline of each adipocyte.

### Immunohistochemistry of white adipose tissue

After deparaffinization, antigen retrieval was performed by cooking tissue slices in citrate buffer for 20 min. Blocking was performed with 10% FCS (Thermo Fisher Scientific, #10270106) and 1% BSA (Sigma-Aldrich, #A9418) in TBS (20 mM Tris, 150 mM NaCl) at RT for 1 h. After blocking, each section was incubated with 50–100 µl primary antibody (Anti-Mouse/Human Mac-2, 6.67 μg/mL, Cedarlane, #CL8942AP or anti-UCP1, Abcam ab10983, 2 μg/mL) which was diluted with PBS (137 mM NaCl, 2.7 mM KCl, 10 mM Na2HPO4, 1.8 mM KH2PO4) containing 1 % BSA at 4°C overnight. After 3 times PBS wash, slices were incubated with 50–100 µl secondary antibody (Alexa FluorTM 647 Goat anti-Rat, 10 μg/mL, Thermo Fisher Scientific, #A21247) in PBS at RT in the dark for 1 h, followed again by 3 washes in PBS. Wheat germ agglutinin (WGA Alexa FluorTM 488 Conjugate, 2 μg/mL, Thermo Fischer Scientific, #W11261) was applied together with secondary antibody. Mounting was performed with ROTI® Mount FluorCare DAPI (Carl Roth).

### Quantification of macrophages

Macrophages in white adipose tissue stained by immunohistochemical staining with anti-MAC-2 antibody were counted manually with the help of software ImageJ. Sections were analyzed by microscope Zeiss Imager. M2 using a 20× objective. All the macrophages in one whole section were counted, the outline of the section was drawn by hand. The final macrophage number of each animal was noted as the number of macrophages in 1 mm^2^ tissue.

### Western blot analysis

White adipose tissue was lysed and homogenized using 500 µl lysis buffer [20 mM Tris-HCl, 1 mM EDTA, 255 mM Sucrose, pH = 7.4, HaltTM Protease & Phosphatase Inhibitor Cocktail (Sigma-Aldrich)] and Qiagen TissueRuptor. Protein concentration was determined using BCA Protein Assay (Thermo Fisher Scientific) and samples were diluted with 4 × Lämmli-buffer (250 mM Tris (pH = 6.8), 8% (v/v) SDS, 20 % (w/v) Glycerol, 0.02 % (v/v) Bromophenol blue, 100 mM DTT). Proteins were separated by SDS-PAGE and semi-dry blotted to nitrocellulose membrane. Before membrane blocking (LI-COR Intercept® Blocking Buffer) RevertTM 700 Total Protein Stain (Licor) was applied according to the manufacturers’ recommendation to determine total protein for normalization. Primary antibody (Anti-UCP1, Abcam ab10983, 1 μg/mL) was incubated in 5% BSA in TBST (2.4% (w/v) Tris, 8.8 % (w/v) NaCl, pH = 7.6, 0.1% Tween) over night at 4°C, followed by 3 × wash in TBST and secondary antibody (IRDye® 800CW Goat anti-Rabbit, 0.1 μg/mL, LI-COR, 926-32211) in blocking solution/0.1 %Tween for 1 h RT in the dark.

### RNA isolation and qPCR analysis

RNA of peripheral white adipose tissue was isolated using Qiagen RNeasy® Lipid Tissue Mini Kit according to the manufacturers’ recommendation. Reverse transcription was performed according to QuantiTect® Reverse Transcription Quick-Start Protocol (Qiagen). Quantitative real time PCR was performed using Platinum® SYBR® Green qPCR SuperMix-UDG (Invitrogen) and StepOne Plus Real-Time PCR Detection System. Calculation of relative gene expression was performed by means of 2^(−ΔCt)^ using *Nudc* as reference gene and normalized to sham-operated animals. Sequences of used primers (Sigma-Aldrich, Thermo Fisher Scientific) are listed in Table 1, Harvard PrimerBank ID (Wang and Seed, 2003; Spandidos et al., 2008; Spandidos et al., 2010) is denoted when applicable.

**TABLE 1:** Primer sequences used for qPCR.

Gene	Primer sequence (5’ to 3’)	Harvard PrimerBank ID	Sigma KiCqStart™ primer batch #
*Adipoq*	fwd: CCA CTT TCT CCT CAT TTC TG		
	rev: CTA GCT CTT CAG TTG TAG TAA C		
*Dgat2*	fwd: GCG CTA CTT CCG AGA CTA CTT	16975490a1	
	rev: GGG CCT TAT GCC AGG AAA CT		
*Fasn*	fwd: GGA GGT GGT GAT AGC CGG TAT	30911099a1	
	rev: TGG GTA ATC CAT AGA GCC CAG		
*Leptin*	fwd: GAG ACC CCT GTG TCG GTT C	6678678a1	
	rev: CTG CGT GTG TGA AAT GTC ATT G		
*Lpl*	fwd: GGG AGT TTG GCT CCA GAG TTT	6678710a1	
	rev: TGT GTC TTC AGG GGT CCT TAG		
*Nudc*	fwd: AGA ACT CCA AGC TAT CAG AC		
	rev: CTT CAG GAT TTC CTG TTT CTT C		
*Pnpla2*	fwd: CAA CCT TCG CAA TCT CTA C		fwd # ST05219114
	rev: TTC AGT AGG CCA TTC CTC		rev # ST05219115
*Pparg*	fwd: AAA GAC AAC GGA CAA ATC AC		
	rev: GGG ATA TTT TTG GCA TAC TCT G		
*Resistin*	fwd: AAG AAC CTT TCA TTT CCC CTC CT	12667798a1	
	rev: GTC CAG CAA TTT AAG CCA ATG TT		

### Statistical analysis

Data are presented as mean ± standard error mean (SEM). Statistical analysis was performed using GraphPadPrism 9. For comparison of two groups unpaired, two-tailed *t*-test or Mann-Whitney test was used. For comparison of groups with two variables 2-way-ANOVA was used followed by Sidak’s multiple comparisons test. Outliers were identified using ROUT test (Figure 3D). *p*-values below 0.05 are indicated by asterisks (*p < 0.05, **p < 0.01, ***p < 0.001). Trends are shown by *p*-values as numbers as indicated.

## Results

### Myocardial ischemia acutely increases circulating NEFA levels and chronically reduces adipocyte size in inguinal white adipose tissue

**FIGURE 1:**
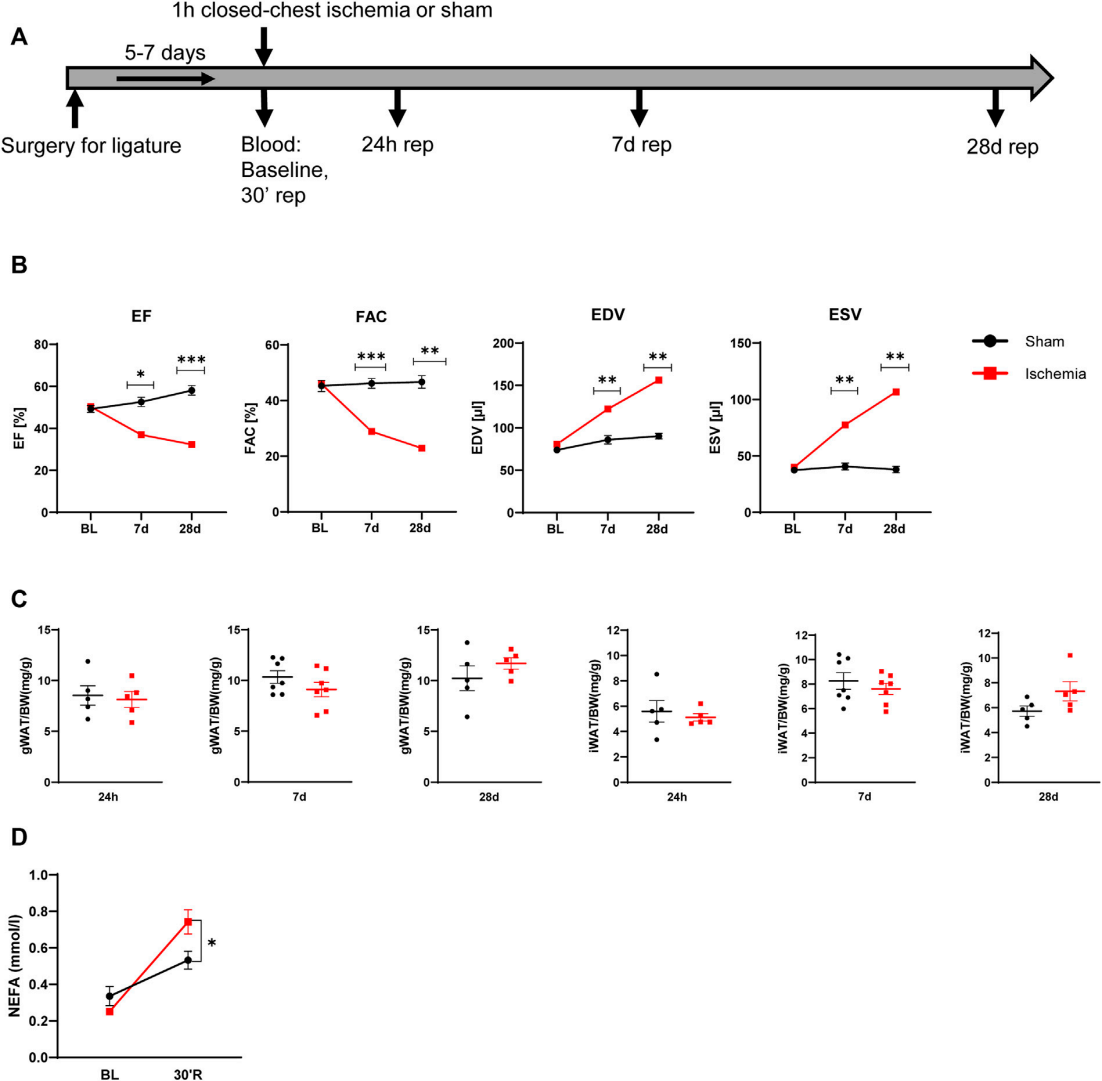
Model of myocardial infarction **(A)** Schematic of used mouse model of myocardial infarction **(B)** Cardiac function and volumes at baseline and after 7 and 28 days reperfusion (*n* = 5) EF: ejection fraction, FAC: fractional area change, EDV: end diastolic volume, ESV: end systolic volume **(C)** iWAT and gWAT weight/body weight ratio (*n* = 5–7) **(D)** Serum NEFA level before ischemia and after 30 min of reperfusion (*n* = 5). Data are mean± SEM, 2-Way-ANOVA **(B and D)** or unpaired, two-tailed *t*-test **(C)**, *p < 0.05, **p < 0.01, ***p < 0.001.

**FIGURE 2:**
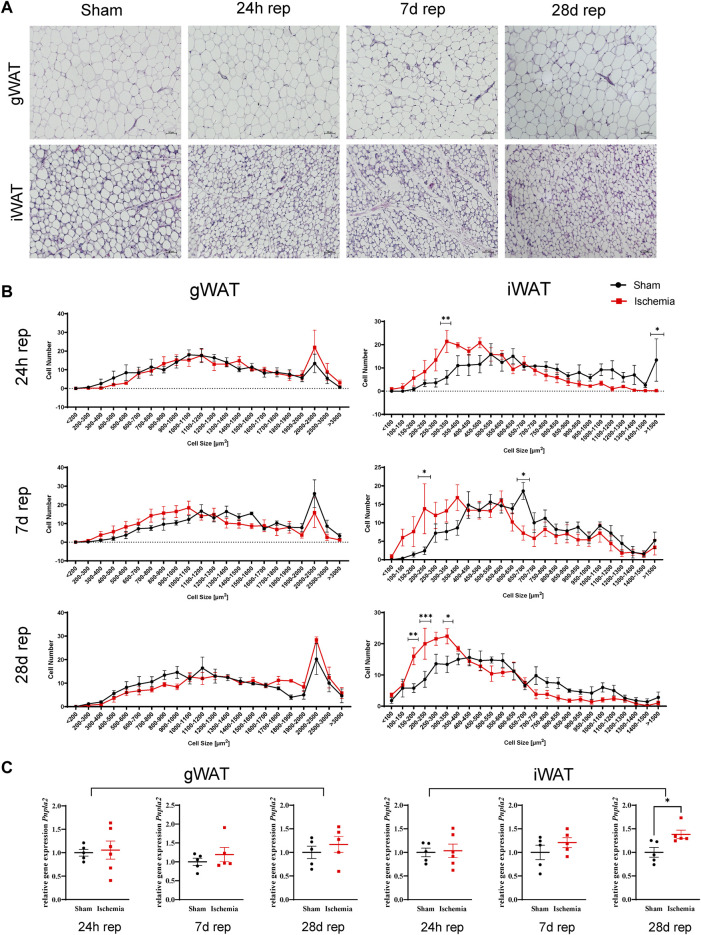
Cardiac I/R reduces adipocytes size predominantly in iWAT **(A)** Hematoxylin and Eosin (H&E) staining of gWAT and iWAT from sham operated animals (24 h) and after 60 min ischemia and 24h, 7, and 28 days of reperfusion. **(B)** Cell size distribution of adipocytes from gWAT and iWAT after 24 h, 7, and 28 days of reperfusion. **(C)** Relative gene expression of *Pnpla2* in gWAT and iWAT after 24 h, 7, and 28 days of reperfusion. *n* = 5–6. Data are mean± SEM, 2-Way-ANOVA **(B)** or unpaired, two-tailed *t*-test **(C)**, *p < 0.05, **p < 0.01, ***p < 0.001.

C57Bl/6J male mice at age of 12 weeks were subjected to 60 min cardiac ischemia or sham operation. Different cohorts of mice were harvested after 24 h, 7 days and 28 days of reperfusion (rep) (Figure 1A). The 28 days rep group, which underwent echocardiography at baseline, day 7 and day 28, showed a significant reduction in systolic pump function (Day 7: EF: 37 ± 3.2%, FAC: 28.9 ± 1.6%, Day 28: EF: 32.3 ± 3.1%, FAC: 22.9 ± 4.2%) and increase in end diastolic and end systolic volumes (Day 7: EDV: 122.3 ± 6 μl, ESV: 77.5 ± 6.7 µl, Day 28: EDV: 156.2 ± 10 μl, ESV: 106.8 ± 11.1 µl) in the ischemia group compared to sham (Figure 1B). The tissue weight to body weight ratios from gonadal WAT (gWAT) and inguinal WAT (iWAT) were unchanged between the two groups (Figure 1C) at all three timepoints. To assess lipolytic activity, serum levels of non-esterified fatty acids (NEFA) were measured before induction of ischemia and after 30′ of reperfusion. The ischemic animals showed a significantly higher 3-fold increase in serum NEFA levels compared to sham operated animals (1.6-fold) (Figure 1D), indicating a stimulation of lipolysis due to cardiac ischemia. To investigate if the lipolytic response after myocardial ischemia induces morphological changes in the different WAT depots, tissue sections of iWAT and gWAT were H&E-stained and adipocyte size was analyzed. While in gWAT no major changes were observed compared to sham controls, iWAT of infarcted mice showed an increase in the number of smaller adipocytes (300–350 μm^2^) and a decrease in the number of bigger adipocytes over 1500 μm^2^ after 24 h of reperfusion. This shift towards smaller adipocyte size was also seen at the later timepoints and was most pronounced 28 days post reperfusion (Figures 2A,B). In line with this, the mean adipocyte size of gWAT was unchanged in comparison to sham controls while iWAT showed a trend towards a reduction of the mean adipocyte size (Supplementary Figure S1), even though statistical significance was not reached. To further investigate the underlying cause for decreased adipocyte size in iWAT after cardiac ischemia, we analyzed gene expression of the main lipolytic enzyme ATGL (*Pnpla2*). This revealed a significant upregulation of *Pnpla2* at the timepoint 28 days of reperfusion only in iWAT, while the gene expression at other timepoints or in gWAT was not affected (Figure 2C). Together, these data validate increased lipolysis in our model of cardiac ischemia and demonstrate changes in adipocyte size in iWAT but not gWAT.

### Myocardial ischemia induces browning of inguinal WAT

**FIGURE 3:**
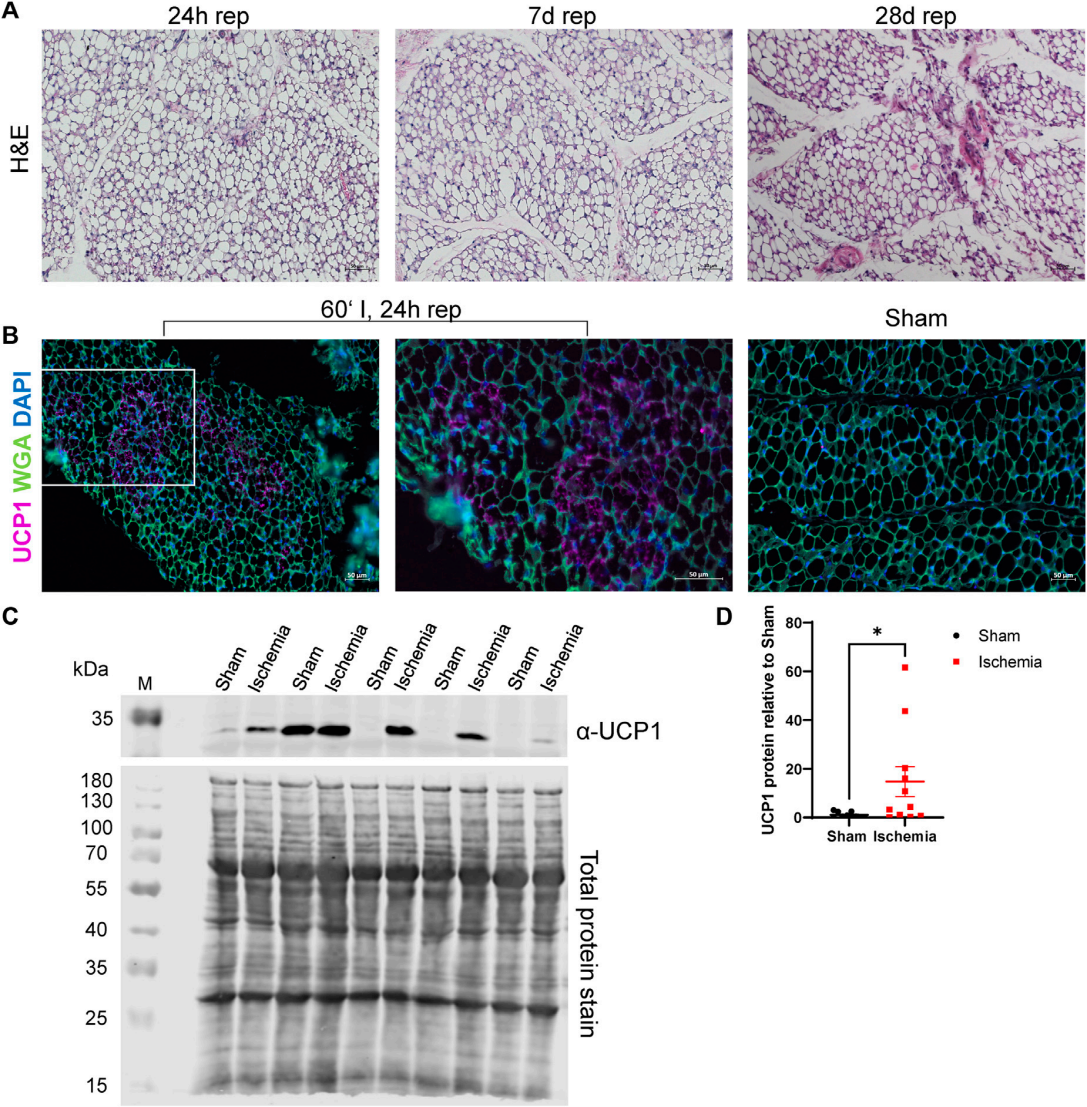
Cardiac I/R induces a brown-like phenotype in iWAT **(A)** H&E staining of iWAT after 60′ ischemia and 24h, 7 days or 28 days of reperfusion. **(B)** Immunofluorescence staining against UCP1 in iWAT of 60‘ ischemia and 24 h reperfusion, 20x (left panel) and 40x (middle panel) magnifications and of Sham/24 h reperfusion (right panel). UCP1: purple, WGA: green, DAPI: blue **(C)** Western blot analysis of UCP1 expression in iWAT after 24 h of reperfusion with corresponding total protein stain of this membrane used for normalization. Uncropped images of these blots are displayed in Supplementary Figure S4. **(D)** Quantification of protein expression of UCP1 relative to Sham, *n* = 13, data are mean± SEM, ROUT test (Q = 1) identified 3 statistical outliers in the „Sham”-group and 2 in the “Ischemia”-group which were excluded, Mann-Whitney test was used for statistical analysis, *p < 0.05.

Next to adipocyte cell size, H&E staining of ischemic animals revealed further morphological abnormalities in iWAT. Adipocytes appeared rather multilocular (Figure 3A), which may indicate “brown-like” or “brite” adipocytes (Barreau et al., 2016). Accordingly, these structures were analyzed for UCP1-expression, which is a common browning marker (Bargut et al., 2017). The staining demonstrated that the striking structures were positive for UCP1 (Figure 3B). We further evaluated UCP1 protein content in samples from 24 h reperfusion animals and indeed found an increase in UCP1 protein levels in ischemic samples compared to sham samples (Figures 3C,D).

### Myocardial ischemia reduces inguinal WAT lipogenesis

**FIGURE 4:**
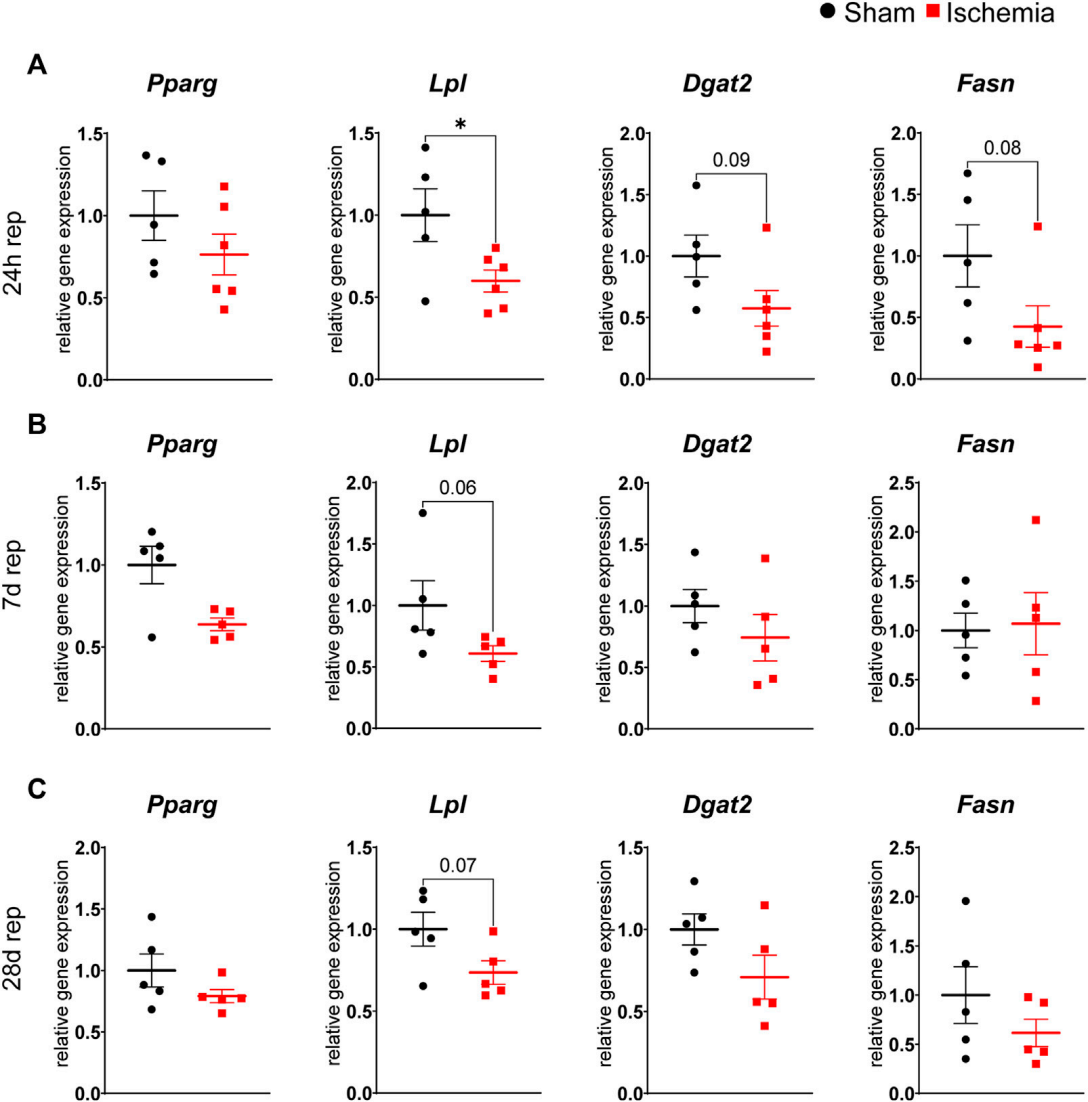
Reduced lipogenesis in iWAT after cardiac I/R Relative gene expression of *Pparg*, *Lpl*, *Dgat2* and *Fasn* in iWAT after 24 h **(A)**, 7 days **(B)** and 28 days **(C)** of reperfusion. n = 5–6, data are mean± SEM, Mann-Whitney test or unpaired, two-tailed *t*-test were used, *p < 0.05.

As myocardial ischemia induced lipolysis, reduced cell size and increased browning of inguinal WAT, we were interested how adipogenesis and lipogenesis of the different WAT depots were affected. While gene expression of the transcription factor PPARγ (*Pparg)* itself was not affected in both depots, downstream genes involved in lipogenesis as lipoprotein lipase (*Lpl*), fatty acid synthase (*Fasn*) and diacylglycerol acyltranferase 2 (*Dgat2*) were either downregulated (*Lpl*) or trended to be downregulated (*Fasn, Dgat2*) after 24 h of reperfusion in inguinal but not in gonadal WAT (Figure 4A and Supplementary Figure S2). This reduced expression of a lipogenic gene signature seemed to be an acute and transient reaction to myocardial ischemia, as expression levels returned to baseline at 7 days and 28 days of reperfusion (Figures 4B,C). Only *Lpl* still trended to be downregulated also at these later timepoints, in line with persistent smaller adipocyte size in subcutaneous WAT.

### Increased infiltration of MAC-2^+^ macrophages in inguinal WAT

**FIGURE 5:**
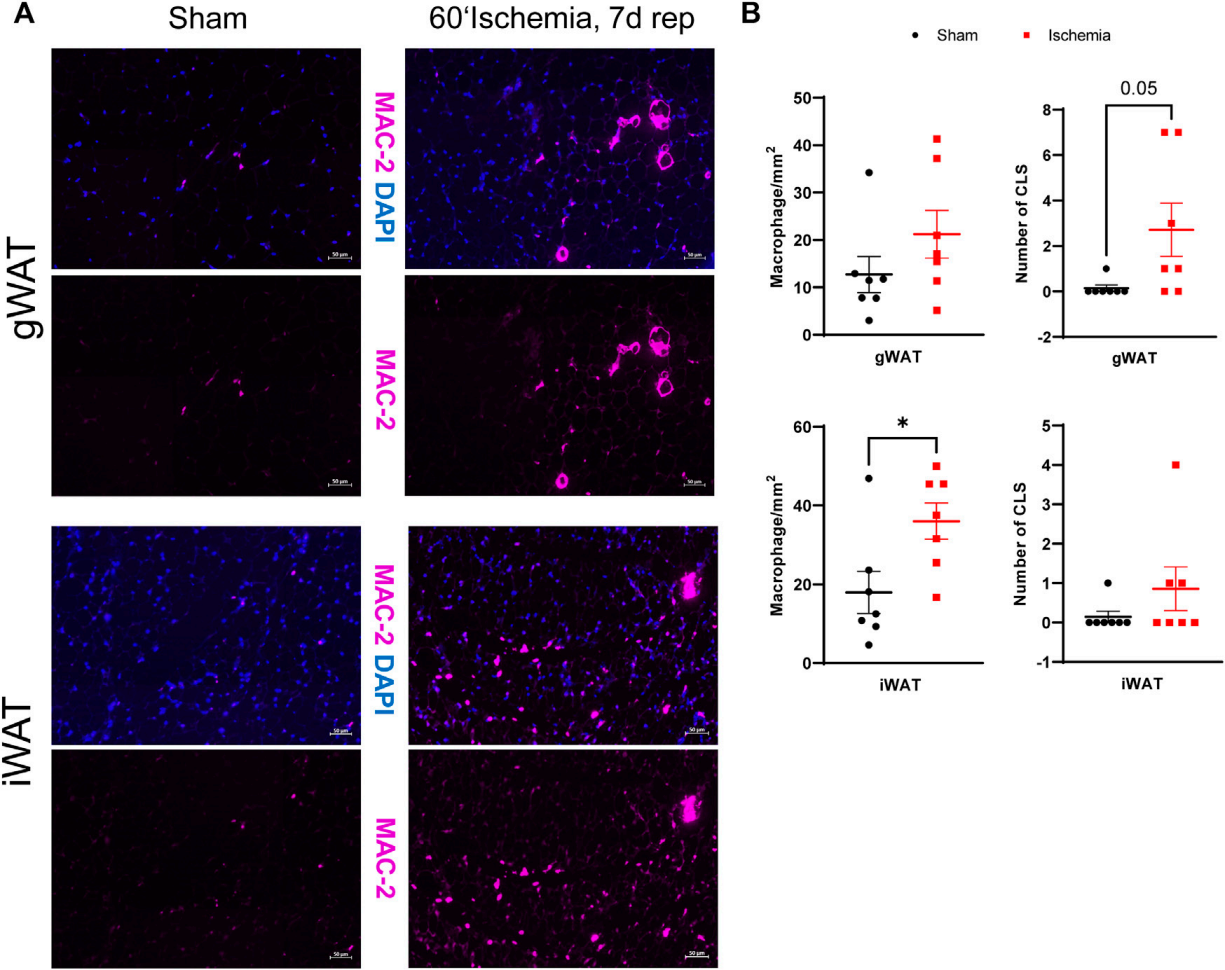
Increased MAC-2+ macrophages in iWAT on day 7 post MI **(A)** MAC-2 immunostaining of gWAT and iWAT from sham operated and ischemic animals after 7 days of reperfusion (MAC-2: purple, DAPI blue). **(B)** Corresponding quantifications of total macrophage numbers and numbers of crown-like structures (CLS) of gWAT and iWAT from sham operated and ischemic animals after 7 days of reperfusion. *n* = 7, data are mean± SEM, Mann-Whitney test or unpaired, two-tailed *t*-test were used, *p < 0.05.

On day 7 of reperfusion, H&E stained sections of WAT contained increased numbers of nuclei from cells of non-adipocyte morphology. Assuming that these cells represented infiltrated immune cells, we stained both WAT depots for MAC-2 (Figure 5A). Quantification of MAC-2 revealed a significant increase of total macrophage numbers in iWAT while they were unchanged in gWAT (Figure 5B). Interestingly, the formation of crown-like structures (CLS), i.e., macrophages surrounding dead or dying adipocytes (Strissel et al., 2007), was significantly enhanced in gWAT but not in iWAT (Figure 5B).

### Myocardial ischemia reduces adipokine expression in inguinal WAT

**FIGURE 6:**
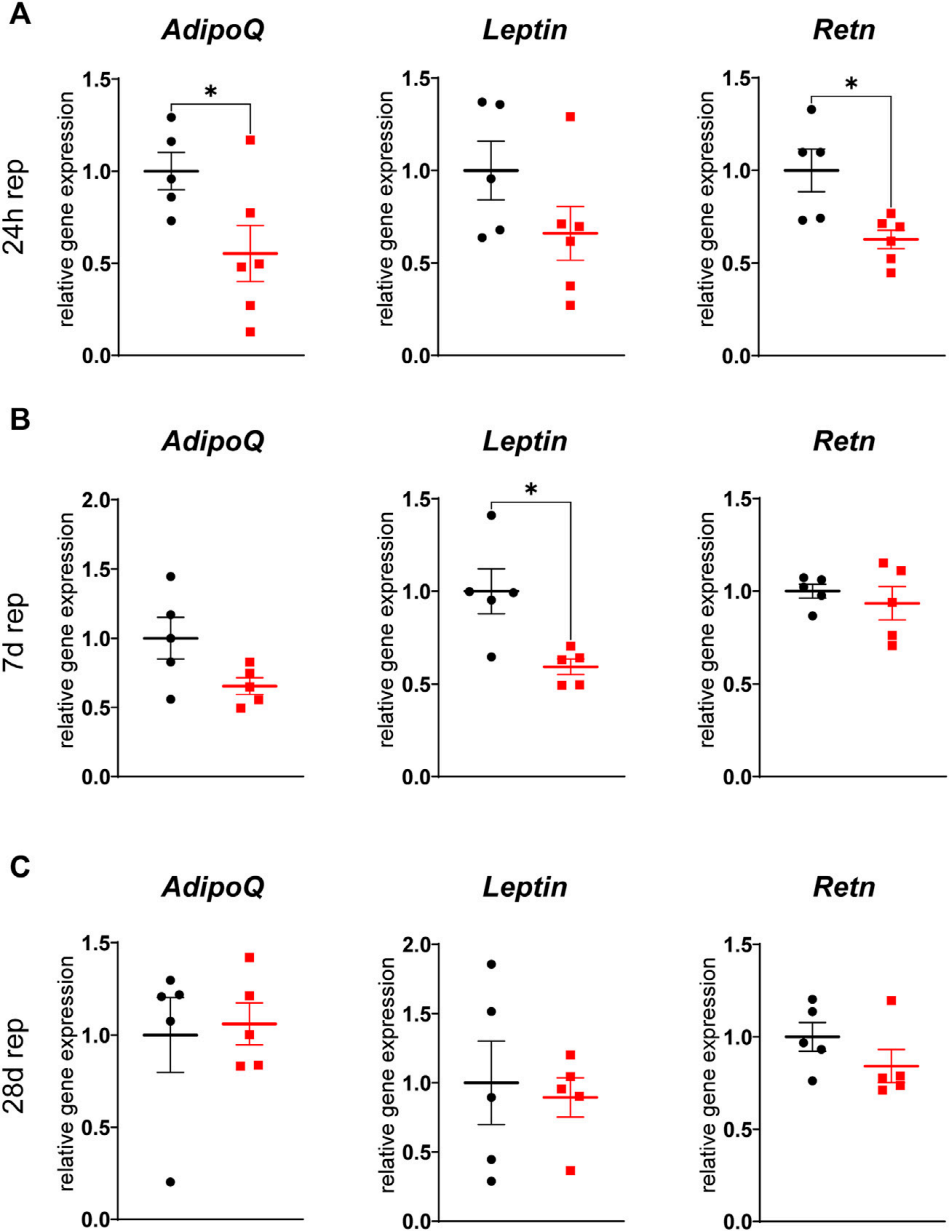
Reduced adipokine gene expression in iWAT after cardiac I/R Relative gene expression of adiponectin *(AdipoQ)*, leptin (*Leptin*) and resistin (*Retn*) in iWAT after 24 h **(A)**, 7 days **(B)**, and 28 days **(C)** of reperfusion. n = 5-6, data are mean± SEM, Mann-Whitney test or unpaired, two-tailed *t*-test were used, *p < 0.05.

Adipose tissue derived factors exert a plethora of systemic functions and several adipokines are known to be involved in post-infarct remodeling. We therefore analyzed gene expression of several adipokines and found three of them, namely adiponectin, leptin and resistin to be differentially expressed. Again, the inguinal WAT was more affected as gene expression of adiponectin and resistin was downregulated in ischemic animals after 24 h of reperfusion and of leptin after 7 days of reperfusion (Figure 6), while expression in gWAT was unchanged (Supplementary Figure S3). These changes were transient, as there was no difference observed after 28 days of reperfusion.

## Discussion

Increasing evidence suggests that targeting adipose tissue and lipolysis is beneficial in various cardiac pathologies (Smeir et al., 2021). However, dissecting physiological and pathogenic functions of lipolysis has proven challenging and requires a better understanding of tissue-specific effects. Here, we used a mouse model of closed chest myocardial ischemia showing activation of lipolysis as measured by increased circulating NEFA levels after 30 min of reperfusion. In line with published data (Oliver, 2014), this increase was transient but interestingly, resulted in chronic alterations in white adipose tissue in a depot-specific manner. Our main findings are:

1) Myocardial ischemia induces alterations of white adipose tissue morphology, gene and protein expression and inflammation up to 28 days after ischemia.
2) The subcutaneous WAT is more susceptible to myocardial ischemia induced changes than the visceral WAT, as we found a reduction in cell size, an increased browning of white adipocytes, a higher macrophage infiltration and a reduction in adipokine gene expression.

The analysis of adipocyte morphology in subcutaneous WAT revealed a sustained reduction of adipocyte size which was associated with an increased gene expression of ATGL at the late timepoint of 28 days of reperfusion. Furthermore, an increased occurrence of multilocular adipocytes which were found to be positive for UCP1 could be observed. Subcutaneous and visceral WAT mainly consist of unilocular white adipocytes with a low number of mitochondria. However, also adipocytes with features of brown adipocytes as multilocularity, high number of mitochondria and UCP1 expression can be found in VAT and SAT. These are called “brown-in-white” or “brite” adipocytes and exhibit these features mainly in response to certain stimuli as cold, catecholamines or exercise (Scheel et al., 2022). In the present study, we detected browning in the subcutaneous depot, in line with observations that browning of white adipose tissue, also in response to other stimuli as cold or β3-agonists, mainly occurs in subcutaneous WAT (Bargut et al., 2017). The fact that the control group underwent anesthesia and sham surgery in the same manner as ischemic animals, showed that the browning effect was indeed due to cardiac ischemia and not to other experimental procedures such as surgical trauma (Longchamp et al., 2016). The most likely explanation for an increased browning in our model of cardiac ischemia and reperfusion is the strong β-adrenergic stimulation, which also activates adipose tissue lipolysis, as seen by an increase in circulating NEFA levels. In addition, also natriuretic peptides, which are elevated after myocardial infarction, were recently identified to promote white adipocyte browning *via* mTOR (Liu et al., 2018). However, the impact of the observed phenomenon on cardiac remodeling is unclear. The implications of browning are generally considered protective in the setting of obesity, as brite adipocytes increase energy expenditure by increasing fatty acid oxidation (Barquissau et al., 2016) and improve insulin sensitivity. However, in the context of energy wasting and hypermetabolic states as cachexia (Tamucci et al., 2018) or burns (Kaur et al., 2021) these effects might not be favorable and therefore aggravate the disease. The mice used in this study were lean C57Bl/6J mice. Interestingly, several hallmarks of adipose tissue which are found in cancer patients, as increased lipolysis, increased browning and reduced lipogenesis (Weber et al., 2022), were also seen in our mouse model of cardiac ischemia/reperfusion. This might indicate that the observed alterations are rather unfavorable, however further studies are needed to elucidate the impact on cardiac remodeling, also in the setting of obesity.

Another finding of the morphological analysis of the different WAT depots, was the increased number of MAC-2+ macrophages in both depots after 7 days of reperfusion. Whereas subcutaneous WAT showed an increase in the overall number of macrophages, visceral WAT contained more crown-like structures, indicating adipocyte apoptosis. Adipose tissue inflammation is a major hallmark of obesity and contributes to adipose tissue and whole-body insulin resistance. Pro-inflammatory M1-like macrophages are the main driver of this low-grade inflammation, which predominantly occurs in visceral WAT depots (Kawai et al., 2021). However, also lipolysis is associated with WAT inflammation (Morris et al., 2011). Lipolytic activation in response to fasting and β3-adrenergic stimulation was shown to increase macrophage infiltration by increasing chemotaxis. Inhibition of lipolysis by knocking out ATGL was able to ameliorate infiltration, indicating a direct link between lipolytic products and macrophage infiltration (Kosteli et al., 2010). Of note, in our mouse model of cardiac ischemia/reperfusion, we found increased macrophage accumulation in subcutaneous WAT despite the fact that circulating free fatty acids were only transiently elevated after ischemia, demonstrating the long-term effects of acute lipolysis activation. Adipose tissue inflammation was also observed in a mouse models of stress induced cardiac hypertrophy and was reduced by ATGL-inhibition (Takahara et al., 2021), indicating that also a more sustained cardiac dysfunction can induce adipose tissue inflammation.

Adipose tissue is known as an active endocrine organ, which secretes a plethora of systemically active protein factors, called adipokines. We showed that cardiac ischemia/reperfusion affects gene expression of several of these adipokines acutely and sustained, only in the inguinal, subcutaneous WAT depot. We found gene expression of adiponectin and resistin to be downregulated after 24 h of reperfusion and of leptin after 7 days of reperfusion. As expression and release of adipokines is closely correlated to adipocyte size (Skurk et al., 2007), this is in line with our observation of reduced adipocyte size in SAT.

Adiponectin levels are known to be transiently reduced after myocardial infarction in humans during the first 72 h after infarction and are nearly normalized again after 7 days (Kojima et al., 2003). Our present data indicate that this reduction might be due to a reduced secretion of adiponectin from the subcutaneous WAT depot. Adiponectin is generally seen as a cardioprotective and anti-inflammatory adipokine, which reduces infarct size in mice (Shibata et al., 2005) and improves cardiac function after experimentally induced myocardial infarction (Shibata et al., 2007). Expression of adiponectin is negatively regulated by insulin, TNFα and dexamethasone (Fasshauer et al., 2002) but also by catecholamines (Fasshauer et al., 2001). It is suggested that adiponectin is a PPARy target gene (Liu and Liu, 2009), which fits to the observation of reduced gene expression of PPARy target genes as *Lpl* in our model.

Next to adiponectin, also the proinflammatory adipokine resistin was downregulated in iWAT after 24 h of reperfusion. Resistin was discovered (Steppan et al., 2001) as a proinflammatory adipokine involved in glucose metabolism and insulin signaling (Gualillo et al., 2007). Several studies show an impact of resistin on cardiac metabolism and function in the context of cardiac hypertrophy (Chemaly et al., 2011; Kang et al., 2011; Zhao et al., 2022), while its role after myocardial infarction is unclear. Plasma levels of resistin in humans were either increased (Korah et al., 2011) or unchanged (Gruzdeva et al., 2014) after MI and high plasma levels are correlated with a higher risk for myocardial infarction (Weikert et al., 2008). In rodent models of cardiac ischemia, resistin either aggravated cardiac dysfunction (Rothwell et al., 2006) or was protective (Gao et al., 2007). Resistin seems to be coupled to lipolytic activity as a model of hemorrhagic shock increased circulating resistin levels, while inhibiting lipolysis attenuated this increase (Raje et al., 2020). Considering this, we were rather surprised to find resistin downregulated in the early phase after cardiac ischemia. Interestingly, PRDM16, a further marker and inductor of browning in white adipose tissue (Seale et al., 2007), was shown to repress resistin gene expression by interacting with C-terminal binding proteins (CtBPs) (Kajimura et al., 2008). As we observe a “brite” phenotype of the inguinal WAT depot after 24 h of reperfusion, this might, at least in part, explain a reduced resistin expression at the same timepoint.

While adiponectin and resistin were altered early after ischemia, leptin was found to be downregulated in SAT after 7 days of reperfusion. In the context of myocardial infarction, leptin was shown to be cardioprotective by direct and indirect effects, for example by protection of cardiomyocytes from apoptosis (Smith et al., 2006; McGaffin et al., 2009) and by improvement of cardiac substrate oxidation (Gava et al., 2021). Circulating leptin levels were found to be elevated in humans in the early phase after myocardial infarction, peaking at day 2 to day 3 (Fujimaki et al., 2001; Khafaji et al., 2012). Our data show a transient decrease at day 7 in leptin gene expression only in subcutaneous WAT, while gene expression in the visceral depot, which has the highest leptin expression in rodents (Trayhurn et al., 1995), is not affected by ischemia/reperfusion. Leptin expression seems to underly a negative feedback regulation (Zhang et al., 1997). Thus, the observed reduced expression at day 7 in our model might be explained by elevated circulating levels before.

Taken together, the here presented study shows a major depot-specific impact of cardiac ischemia and reperfusion on the subcutaneous WAT, which occurs not only acutely after a strong induction of lipolysis *via* catecholamines, but also more prolonged during the post-ischemic remodeling process. These findings contribute to a better understanding of the heart–adipose tissue crosstalk in cardiac I/R and might open up new therapeutic strategies in the prevention and treatment of post-infarction heart failure.

## Data Availability

The original contributions presented in the study are included in the article/Supplementary Material, further inquiries can be directed to the corresponding author.

## Abbreviations

Brite, Brown-in-white; BW, Bodyweight; CLS, Crown-like structure; EDV, End diastolic volume; EF, Ejection fraction; ESV, End systolic volume; FAC, Fractional area change; FFA, Free fatty acids; I/R, Ischemia/reperfusion; NEFA, Non-esterified fatty acids; SAT, Subcutaneous adipose tissue; TAG, Triacylglycerides; WAT, White adipose tissue; VAT, Visceral adipose tissue
