# Supplementary material for: Cardiac ischemia modulates white adipose tissue in a depot-specific manner
Source: Front Physiol. 2022 Oct 28;13:1036945. doi: 10.3389/fphys.2022.1036945 (PMC9649620; doi:10.3389/fphys.2022.1036945)
Supplement: Supplementary file 1 [file DataSheet1.pdf]

SUPPLEMENTARY MATERIAL

Cardiac ischemia modulates white adipose tissue in a depot-specific manner

Luzhou Wang, Heba Zabri, Simone Gorressen, Dominik Semmler, Christian Hundhausen, Jens W. Fischer, Katharina Bottermann

Institute for Pharmacology, Medical Faculty and University Hospital Düsseldorf, Heinrich-Heine-University Düsseldorf, Postfach 101007, 40001 Düsseldorf, Germany

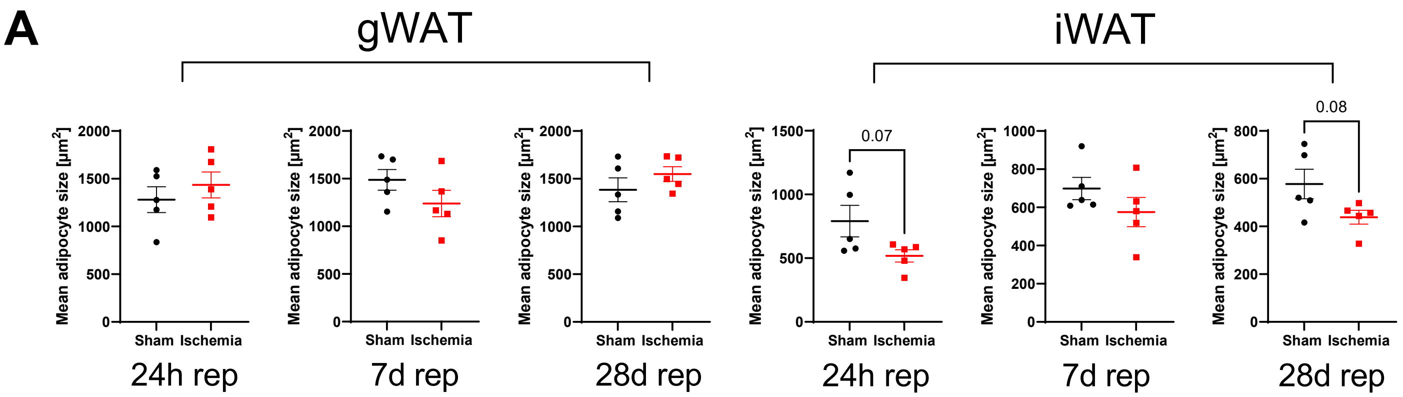

Supplementary Figure S1: (A) Mean adipocyte cell size of adipocytes from iWAT and gWAT after 24h, 7d and 28d of reperfusion. n=5, Data are mean +/-SEM, unpaired, two-tailed t-test.

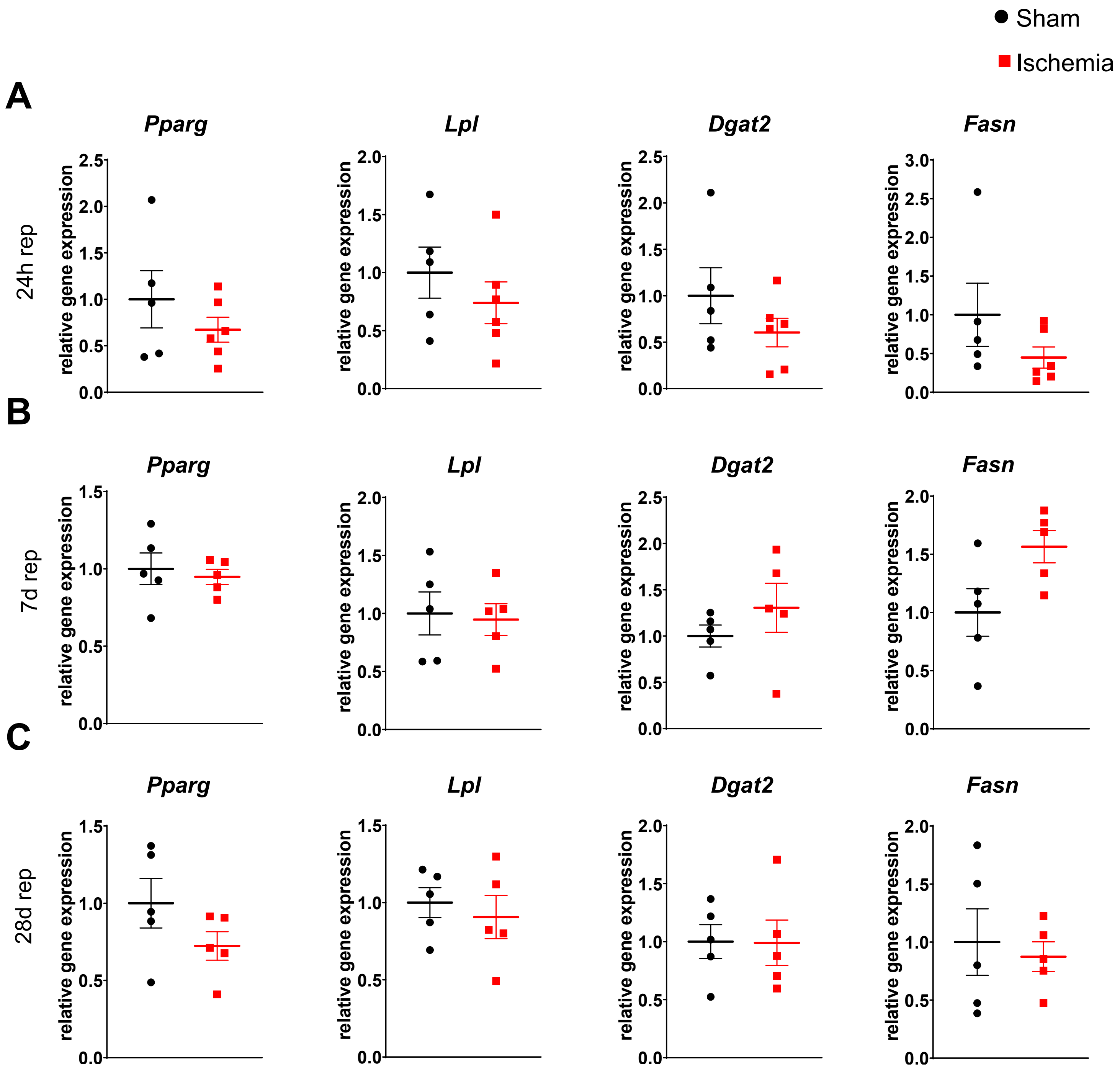

Supplementary Figure S2: Lipogenesis in gWAT following cardiac ischemia
Relative gene expression of *Pparg*, *Lpl*, *Dgat2* and *Fasn* in gWAT after 24h (A), 7d (B) and 28d (C) of reperfusion. n=5-6, data are mean +/-SEM, Mann-Whitney test or unpaired, two-tailed t-test were used.

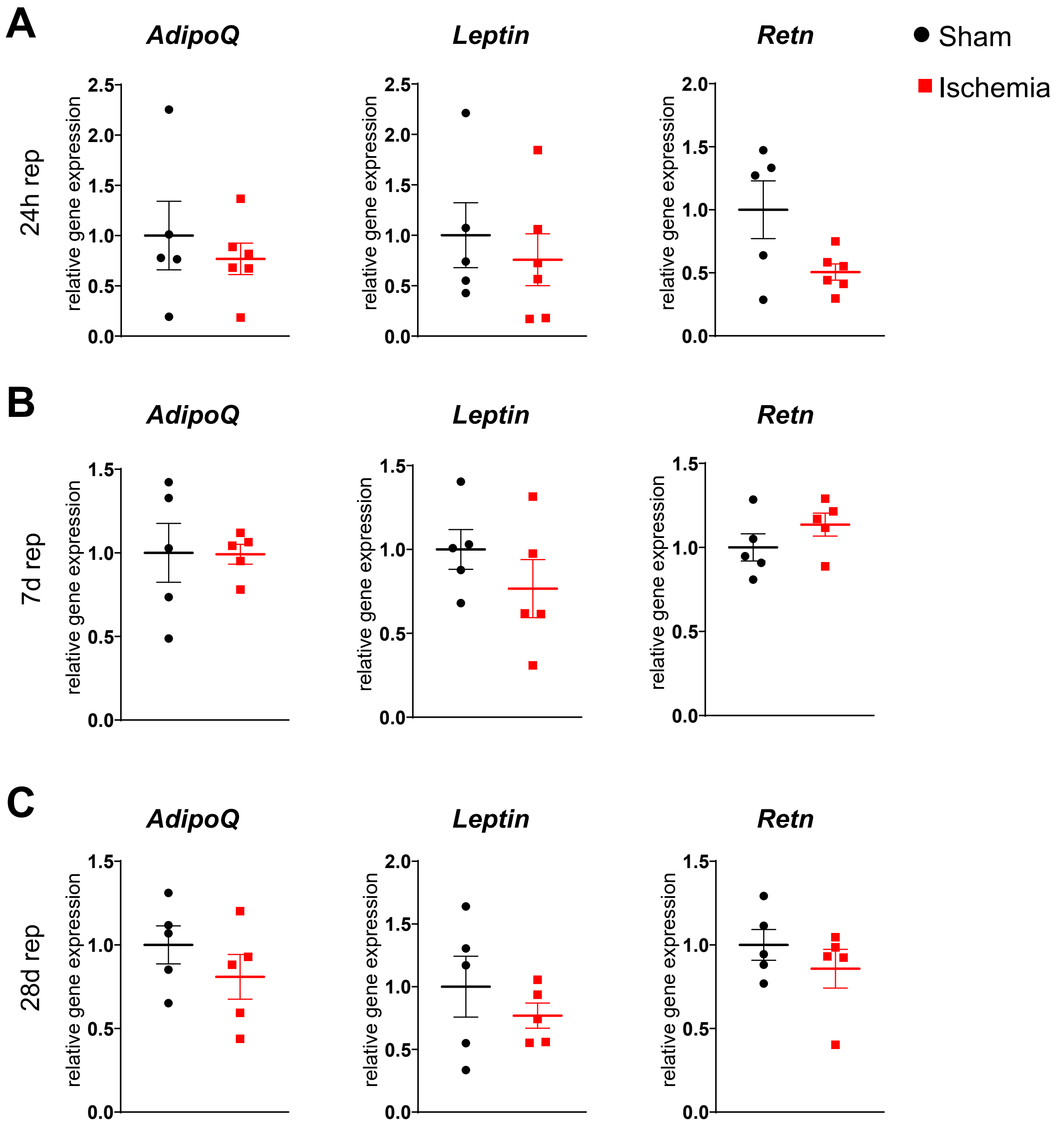

Supplementary Figure S3: Adipokine expression in gWAT after cardiac ischemia
Relative gene expression of adiponectin (*AdipoQ*), leptin and resistin (*Retn*) in gWAT after 24h (A), 7d (B) and 28d (C) of reperfusion. n=-5-6, data are mean +/-SEM, Mann-Whitney test or unpaired, two-tailed t-test were used.

A

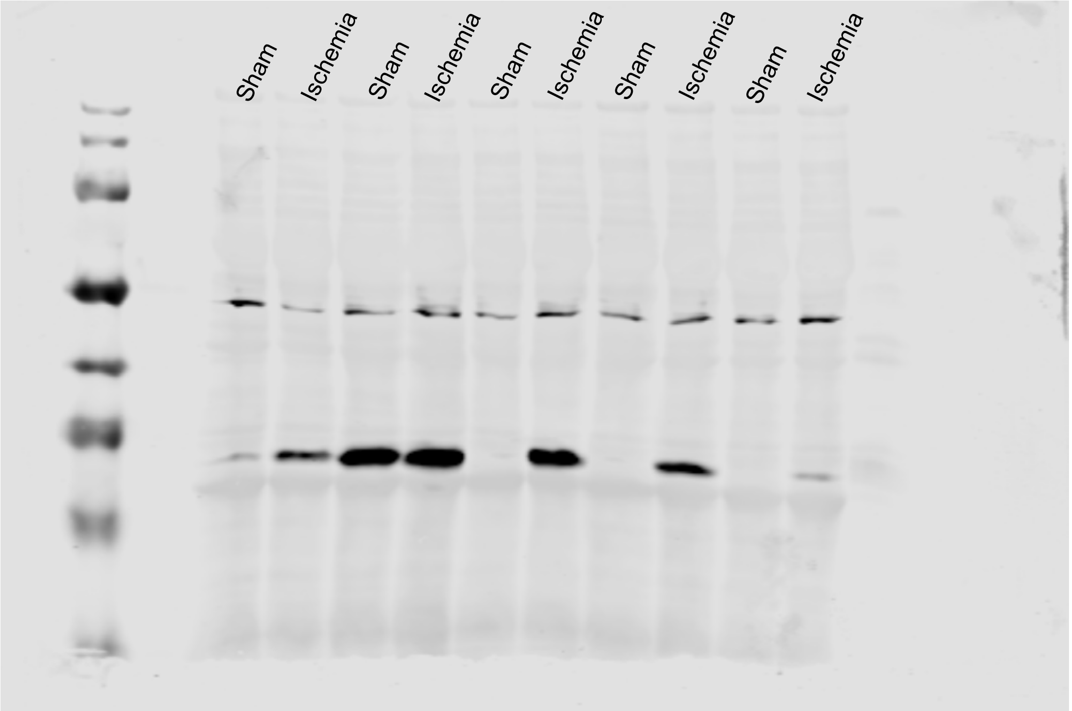

B

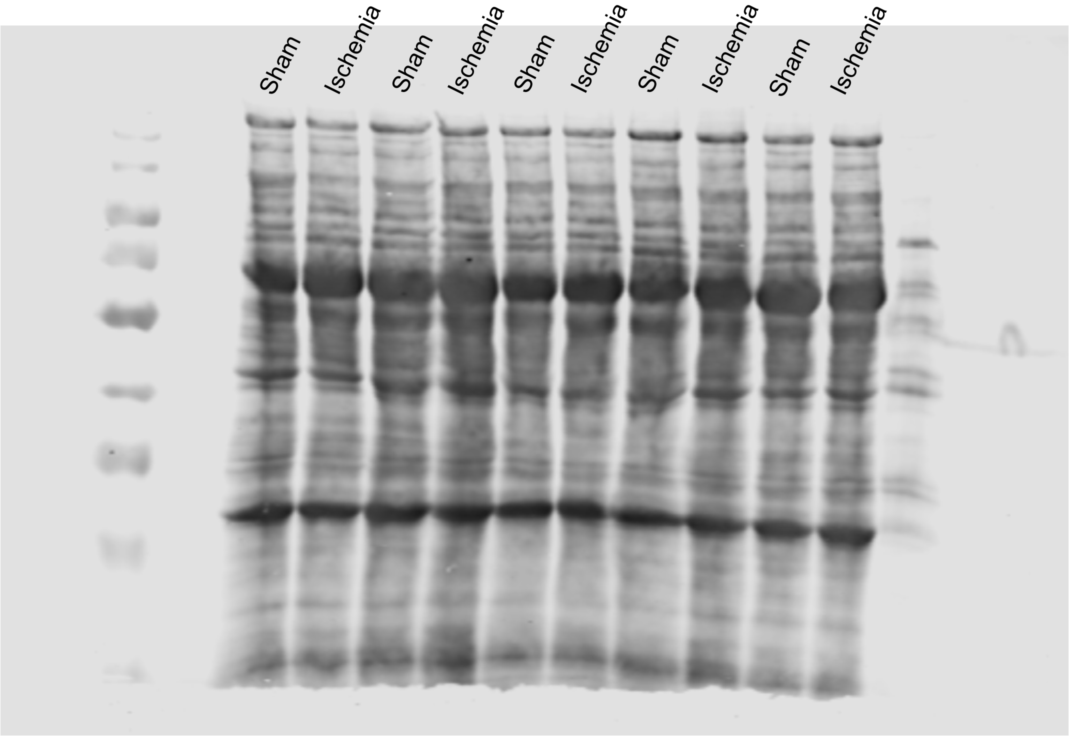

Supplementary Figure S4: (A) Uncropped western blot analyzed for α-UCP1 from Figure 3. (B) Total protein stain of this western blot used for normalization.
